# HPV+ Oropharyngeal Cancer in Waldenström Macroglobulinemia

**DOI:** 10.7759/cureus.38965

**Published:** 2023-05-13

**Authors:** Shahm Raslan, Mursalin M Anis

**Affiliations:** 1 Hematology and Oncology, Florida International University, Herbert Wertheim College of Medicine, Miami, USA; 2 Otolaryngology, University of Miami Miller School of Medicine, Miami, USA

**Keywords:** marijuana use, oral and oropharyngeal cancer, head and neck oncology, waldenstrom macroglobinaemia, human papillomavirus (hpv)

## Abstract

We present a case of squamous cell carcinoma (SCC) in the setting of Waldenström macroglobulinemia (WM). A 68-year-old male and daily marijuana smoker with recently diagnosed WM presented via telemedicine in 2020 for a progressively worsening sore throat and unintentional weight loss. Immunotherapy for WM was delayed due to the COVID-19 pandemic. In the clinic, examination revealed an indurated, tender midline mass at the base of the tongue, not limiting tongue mobility. The left level-II and right level-III lymph nodes were enlarged. The oropharyngeal lesion was biopsied, and pathology was consistent with human papillomavirus-positive (HPV+) SCC. Four cycles of concurrent chemotherapy and radiation for SCC were administered without delay, with an initial response. However, on surveillance, metastases to the brain and lungs were detected, and the patient was placed on palliative treatment as he did not meet eligibility for a clinical trial due to his WM. Concurrent WM and HPV+ SCC may have a worse prognosis, due to disease progression and reduced therapeutic options.

## Introduction

Waldenström macroglobulinemia (WM) is a rare indolent lymphoplasmacytic lymphoma, a non-Hodgkin's lymphoma (NHL). Lymphomas are the second most common malignancy of the head and neck, with 65-90% of head and neck lymphomas being NHL [1]. WM may present as lymphoid lesions in the head and neck, although WM remains 50-fold less common than primary oropharyngeal cancer [2-3]. We present a case of squamous cell carcinoma (SCC) in the setting of WM, for which immunotherapy for WM was deferred due to the COVID-19 pandemic.

## Case presentation

A 68-year-old male maintenance worker with a history of eyelid cancer and recently diagnosed WM presented with a complaint of a sore throat for two months. He had not received the rituximab prescribed for WM due to difficulties associated with the COVID-19 pandemic. He reported pain with tongue protrusion, swallowing, throat palpation, and intermittent left-sided otalgia. His throat pain reportedly worsened as the days progressed, especially with citrus drinks or cold drinks. He also reported fatigue, an unintentional six-pound weight loss, and a deepening voice. He reported smoking four to five hits of marijuana daily and previous tobacco smoking of less than one pack per day, with cessation 35 years ago.

**Figure 1:**
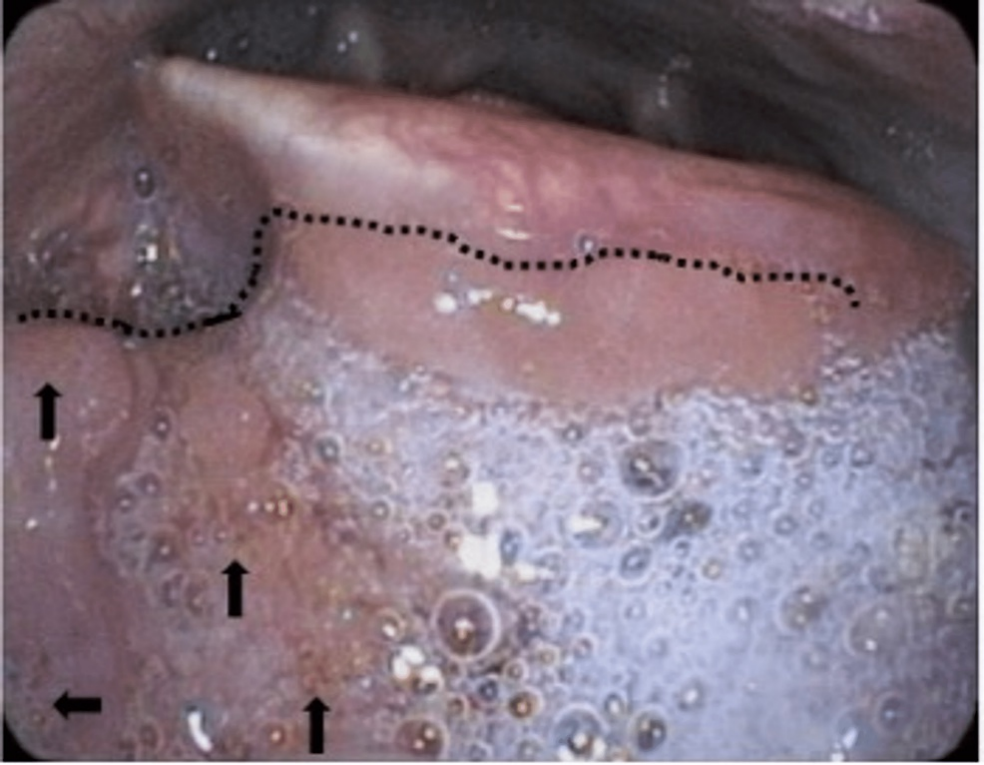
Tongue base mass (dotted lines) is similar in appearance to prominent lingual tonsils but with tortuous and speckled microvasculature (arrows).

Due to concerns about the patient’s history from the telemedicine visit, he was immediately scheduled to be seen in clinic. A complete head and neck exam revealed an indurated, tender midline mass on palpation of the base of the tongue without limitations to the tongue’s mobility. He had a palpable right-sided 2 cm level-III lymph node and a left-sided level-II 3 cm lymph node. Flexible laryngoscopy was performed and was notable for a tongue base mass similar in appearance to a prominent lingual tonsil but with tortuous and speckled microvasculature (Figure 1).

**Figure 2:**
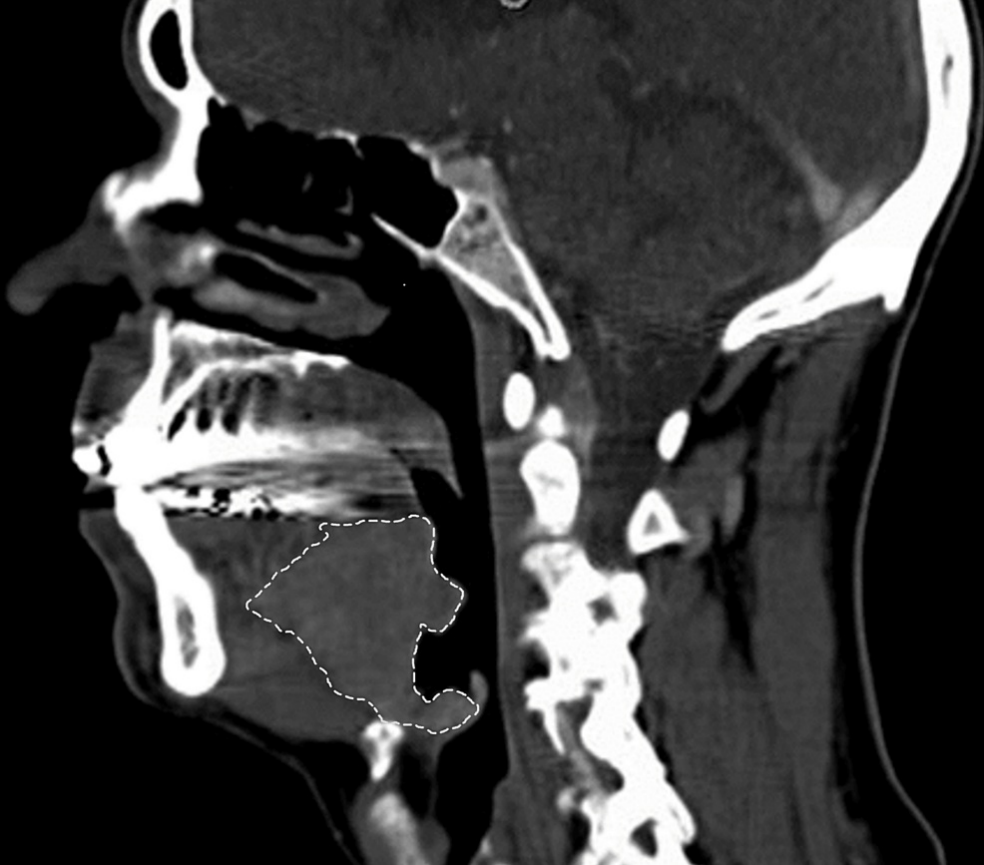
Computed tomography of the neck with contrast revealed a large infiltrative lingual mass suspicious for carcinoma measuring approximately 4.1 x 3.4 x 3.6 cm (dotted line). The mass can be seen involving the lingual surface of the epiglottis and inseparable from portions of the hyoid bone.

Computed tomography of the neck with contrast revealed a large infiltrative lingual mass suspicious for carcinoma (Figure 2).

The patient underwent an office-based biopsy of the base of the tongue lesion, and pathology revealed human papillomavirus-positive (HPV+) SCC. DNA of HPV type 16 was detected. Because of his WM and treatment with rituximab, he did not meet the selection criteria for a clinical trial examining a novel combination of immunotherapy and chemotherapy for HPV+ SCC. Immunotherapy for WM continued to be deferred during the COVID-19 pandemic, though he received four cycles of chemoradiation without delay, with an adequate response, and without reported complications. He showed no signs of oropharyngeal disease at his last follow-up but unfortunately developed metastases to his brain and lungs and was placed on palliative treatment.

## Discussion

Oropharyngeal cancer may present as a nonhealing red or white lump or ulcer. Particular attention should be given to regional lymph nodes during a thorough head and neck exam. When there is a history of heavy smoking or alcohol consumption, a flexible laryngoscopy helps to evaluate the oropharynx, hypopharynx, and larynx to identify the presence and superficial extent of any lesion. Clinically suspicious lesions with aberrant microvasculature should be biopsied. Even though HPV infection of the oral cavity has a bimodal age distribution at ages 30-34 and ages 60-64, HPV+ oropharyngeal SCC has a peak incidence at ages 60-64 [4]. HPV+ oropharyngeal cancer should be high in the differential in patients who use marijuana daily and present with oropharyngeal lesions.

WM may present with symptoms of hyperviscosity and may also present with weight loss, neuropathy, and fatigue, all of which may be alleviated by cannabis use. In hematologic malignancies, cannabinoids may activate pathways of programmed cell death and may promote response to chemotherapy [5-6]. In HPV+ SCC in particular, cannabinoids activate the p38 MAPK pathway and stimulate HPV+ disease progression in the head and neck [7]. Both cannabis use and HPV are on the rise in the US. HPV is rarely associated with head and neck lymphomas [2], although this is an area of ongoing research. Vaccination can reduce the risk of infection from high-risk HPV types by 46-80% [8]. Vaccination programs targeting all segments of the population remain the most effective at preventing HPV infection [9-10].

## Conclusions

WM can present with head and neck lesions that present similarly to primary oropharyngeal SCC. Cannabinoids may have beneficial effects in the setting of hematologic malignancies, yet they are known to promote HPV+ SCC. Despite underlying hematologic malignancy, HPV+ oropharyngeal cancer should be high in the differential in patients who use marijuana daily and present with oropharyngeal lesions. Molecular links between WM and HPV+ oropharyngeal SCC are limited and must be further explored. HPV+ SCC in patients with WM may have a worse prognosis due to disease progression and reduced therapeutic options.
